# The complete mitochondrial genome of *Pericapritermes nitobei* (Isoptera: Termitidae)

**DOI:** 10.1080/23802359.2018.1481793

**Published:** 2018-06-26

**Authors:** Yiyuan Liao, Shengli Lu, Haihong Chen, Dayu Zhang

**Affiliations:** a The Key Laboratory for Quality Improvement of Agricultural Products of Zhejiang Province, College of Agricultural and Food Science, Zhejiang A&F University, Linan, China;; b Yuyao Housing Safety Management and Service Center, Yuyao, China;; c Ningbo Housing Safety Management and Service Center, Ningbo, China

**Keywords:** *Pericapritermes nitobei*, Isoptera, mitochondrial genomes

## Abstract

The complete circular mitochondrial genome of a higher termite *Pericapritermes nitobei* has a length of 15,224bp and encodes 37 genes including 13 protein-coding genes (PCGs), 22 transfer RNA (tRNA), 2 ribosomal RNA (rRNA) and a non-coding control region (D-loop). Protein coding genes (PCGs) in this circular mitogenome start with standard ATN initiation codons and end with complete termination codons TAN except for *cox2* and *nad5* genes with an incomplete stop codon T. The percentage of A and T (67.49%) is higher than that of G and C (32.51%). The phylogenetic tree revealed that mitogenomes of Pericapritermes formed one clade. The tree also revealed that *Pericapritermes dolichocephalus* and *Pericapritermes latignathus* constituted a sister group to *P. nitobei*. The date here provide a resource for genetics and evolution analysis within termites especially Pericapritermes genus.

Pericapritermes genus contains 33 species and mainly distributes in tropical and subtropical regions (Tan and Yan, 2013). As typical species of higher termites, the mitochondrial genomes of Pericapritermes are applied for termites evolutionary analysis (Bourguignon et al. 2015, 2017). *Pericapritermes nitobei* was first described as *Eutermes nitobei* and Krishna revised the species to *P. nitobei* (Krishna et al. 1968). Although there are some studies on the mitochondrial genes of *P. nitobei* such as *rrnL*, *rrnS* and *cox2* (Ohkuma et al. 1999, 2004; Bujang et al. 2014), there is no available information about its complete mitochondrial genome. The present study was the first report on the complete mitochondrial genome sequences of *P. nitobei.*

Specimens were collected from Ningbo city, China and kept in the insect lab at Zhejiang A & F University with Accession number NB0024-TT-4. The entire mitochondrial genome sequence of *P. nitobei* was 15,224 bp in length, including 13 protein-coding genes, 22 tRNA genes, 2 rRNA genes (*rrnL* and *rrnS*) and 1 non-coding control region (D-loop). Most of these genes were located on the H-strand except for *nad1, nad4, nad4l, nad5* and 8 tRNA genes (*trnQ, trnC, trnY, trnF, trnH, trnP, trnL1* and *trnV*). The genetic compositions and coding sequences are similar to other termites (Zhao et al. 2016; Hervé and Brune 2017).

The overall base compositions of *P. nitobei* mitochondrial genome had a high A/T tendency. The A + T content of *P. nitobei* was 67.49%, higher than that of G + C (32.51%). The mitochondrial genome of *P. nitobei* included intergenic spacers and overlapping regions. The intergenic spacer sequences were on 22 regions ranging in size from 1 to 17 bp. The overlapping sequences varied from 1 to 44 bp in five areas.

The mitochondrial genome of the *P. nitobei* contained 13 protein-encoding genes, with the total length of 11,144 bp. All protein-coding genes (PCGs) start with standard ATN initiation codons and end with complete termination codons TAN except for *cox2* and *nad5* genes with an incomplete stop codon T. There were 22 tRNA genes in the mitochondrial genome of *P. nitobei*. Except for the tRNA-Ser, which lacked the dihydrorubamide (DHU) arm, the other tRNAs have the classical cloverleaf structures.

The control region (D-loop) with 336 bp in size is located between *rrnS* and *trnI* gene. The content of A + T in control region was 70.54%, and its content was significantly higher than that of the whole mitochondrial genome.

*Pericapritermes nitobei* mitochondrial genome has two ribosomal RNAs, *rrnL* and *rrnS. rrnL* is located between *trnL1* and *trnV* with the length of 1368 bp and *rrnS* is located between *trnV* and the control region with 806 bp in size.

A phylogenetic tree among Pericapritermes was constructed using a dataset which contained all the nucleotide sequences of PCGs of Pericapritermes mitochondrial genes. *Microcerotermes crassus* and *Termes fatalis* (Termitidae) were set as outgroups. Two major clades were formed, one was *M. crassus* and *T. fatalis*, the other was the remaining species (Pericapritermes) (Figure 1). The tree also revealed that *P. dolichocephalus* was closest to *P. latignathus* and constituted a sister group to *P. nitobei*.

**Figure 1.**
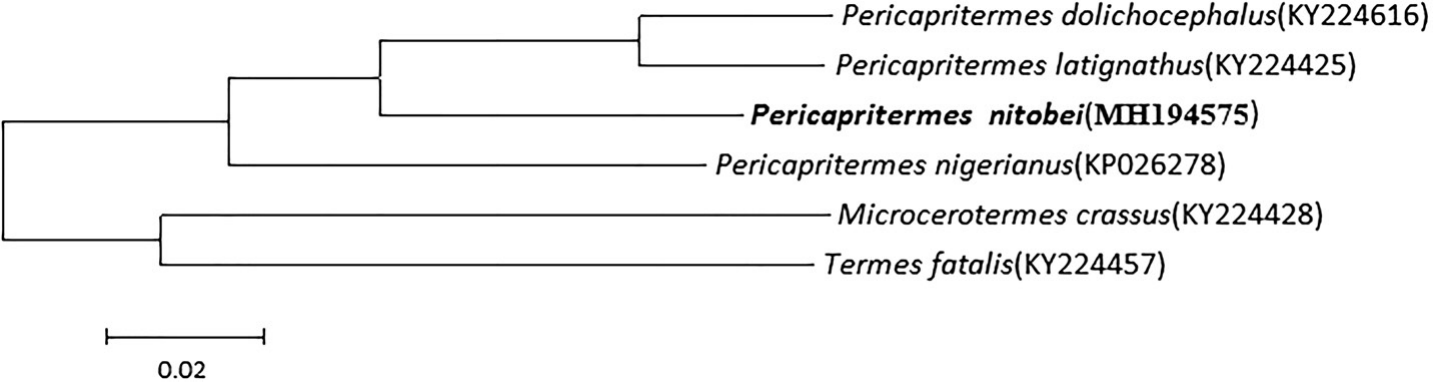
Maximum Likelihood phylogenetic tree of selected termite mitogenomes including Pericapritermes. The phylogenetic tree was constructed using all 13 PCGs. Leaf names were presented as species names and GenBank accession number.

## Nucleotide sequence accession number

The complete genome sequence of *P. nitobei* has been assigned GenBank accession number (MH194575).
